# ISYNA1: An Immunomodulatory-Related Prognostic Biomarker in Colon Adenocarcinoma and Pan-Cancer

**DOI:** 10.3389/fcell.2022.792564

**Published:** 2022-02-14

**Authors:** Zeming Jia, Xiaoping Wan

**Affiliations:** Department of General Surgery, Xiangya Hospital, Central South University, Changsha, China

**Keywords:** TCGA, ISYNA1, colon adenocarcinoma, prognostic biomarker, macrophages, immunosuppressive microenvironment

## Abstract

**Background:** Colon adenocarcinoma (COAD) is a common digestive system tumor in the world. However, the role and function of ISYNA1 (inositol-3-phosphate synthase 1) in COAD remain unclear. We aim to explore the role of ISYNA1 in pan-cancer, especially in COAD.

**Methods:** The expression, clinical characteristic, and prognosis of ISYNA1 in pan-cancer were evaluated using the TCGA (the Cancer Genome Atlas), GTEx (the Genotype-Tissue Expression), and CCLE (Cancer Cell Line Encyclopedia). Pathway enrichment analysis of ISYNA1 was conducted using the R package “clusterProfiler.” We analyzed the correlation between the immune cell infiltration level and ISYNA1 expression using two sources of immune cell infiltration data, including the TIMER online database and ImmuCellAI database.

**Results:** ISYNA1 was highly expressed in COAD and other cancer types compared with respective normal tissues. High ISYNA1 expression predicted poorer survival in COAD. We also found that ISYNA1 expression was positively correlated with the infiltration level of tumor-associated macrophages and tumor-associated fibroblasts in COAD.

**Conclusion:** In conclusion, our findings revealed ISYNA1 to be a potential prognostic biomarker in COAD. High ISYNA1 expression indicates the immunosuppressive microenvironment.

## Introduction

Colon adenocarcinoma (COAD) is a global common gastrointestinal tumor, which presents a fatal factor to the health of humans (Wei et al., 2020). The early stage of COAD has no obvious symptoms, leading to the diagnosis of many patients at an advanced stage (Zhou W et al., 2020). Thus, it is pressing to explore the potential mechanisms and identify crucial biomarkers for diagnosis, prognosis, and treatment in COAD.

A body of work have found that the tumor microenvironment (TME), especially the tumor immune microenvironment (TIME), plays an important role in the development of COAD and weakens the response of COAD patients for treatment (Lin et al., 2020). The immune system in the TME was always rebuilt by tumor cells and accelerated tumor progression. For example, tumor-associated macrophages (TAMs) were rebuilt directly or indirectly by tumor cells, and in turn play an immunosuppressive and tumor-promoting role in the process of tumor development (Choi et al., 2018; Qiu et al., 2018; Deepak et al., 2020; Liu et al., 2021). Except for the immune cells, stromal cells in TME were also an accomplice in tumor progression. Stromal cells like cancer-associated fibroblasts (CAFs) could release cytokines, such as TGFB1, IL6, and IL8, and accelerate tumor progression.

ISYNA1 (inositol-3-phosphate synthase 1) encodes the rate-limiting enzyme inositol-3-phosphate synthase. Until now, there are few studies on ISYNA1 in tumor research. In our study, we comprehensively analyzed the role of ISYNA1 using multi-omics data from the TCGA database for 33 cancers, including expression, clinical features, prognostic values, DNA methylation, copy number alteration (CNA), and mutation status of ISYNA1. The correlation between ISYNA1 expression and immune cell infiltration was further assessed. The high expression of ISYNA1 predicted the immunosuppressive TIME, which may lead to poorer survival of tumor patients with a high ISYNA1 expression in COAD. Therefore, targeting ISYNA1 may be a potential cancer therapy method in COAD.

## Materials and Methods

### RNA Extraction and qRT-PCR

Tumor and adjacent normal tissues of COAD, rectum adenocarcinoma (READ), and liver hepatocellular carcinoma (LIHC) were obtained from the Department of General Surgery, Xiangya Hospital. The research protocols were approved by the Research Ethics Committee of Xiangya Hospital. All participants gave written consent of their tissue samples and medical information to be analyzed for scientific research. The total RNA of tissues was isolated and purified by AG RNAex Pro Reagent, AG21101 (Accurate Biology, China), following the manufacturer’s instructions. Quantitative PCR (qPCR) analysis of samples was performed using EvoM-MLV RT Kit, AG11711 (Accurate Biology, China) following the manufacturer’s instructions. ACTB1 was used as the internal control. The primers were designed as follows:

ACTB1:

Forward 5′-GGC​ATT​CAC​GAG​ACC​ACC​TAC-3′.

Reverse 5′-CGA​CAT​GAC​GTT​GTT​GGC​ATA​C-3′.

ISYNA1:

Forward 5′-CAG​CAC​CGG​GTT​TTT​GTG​G-3′.

Reverse 5′-TCC​TTA​GAG​CGG​AAC​TGC​AAT-3′.

CD274 (PDL1):

Forward 5′-TGG​CAT​TTG​CTG​AAC​GCA​TTT-3′.

Reverse 5′- TGC​AGC​CAG​GTC​TAA​TTG​TTT​T-3′.

### Data Collection

The expression profiles and clinical information of the Cancer Genome Atlas (TCGA) and Cancer Cell Line Encyclopedia (CCLE) were downloaded from the UCSC Xena (https://xenabrowser.net/datapages/) database. The infiltration level of different immune cells in TCGA was downloaded from the ImmuCellAI database (http://bioinfo.life.hust.edu.cn/ImmuCellAI#!/).

### Online Website

The pan-cancer expression of ISYNA1 in TCGA cohort was performed using the TIMER2 database (http://timer.comp-genomics.org/). The cBioPortal database was used to analyze the CNA and mutation status of ISYNA1. The correlation between ISYNA1 expression and infiltration level CAFs/TAMs was performed using the TIMER2 database.

### Correlation and Gene Set Enrichment Analysis

The association between the ISYNA1 expression level and all mRNAs was analyzed using TCGA RNAseq data, and Pearson’s correlation coefficient was calculated. The mRNAs correlated with ISYNA1 (*p* < 0.05) were ranked and subjected to the GSEA. The analysis was conducted using the R package “clusterProfiler.”

### Relationship Between ISYNA1 Expression and TME

The TME gene sets were downloaded, and the scores of each gene set in TCGA pan-cancer were calculated using the method from this published study (Zeng et al., 2019).

### Correlation Between ISYNA1 Expression and Immune Cell Infiltration

First, the correlation between ISYNA1 expression and infiltration level CAFs/TAMs was performed using the TIMER2 database. Second, we downloaded the immune cell infiltration data of TCGA pan-cancer from the ImmuCellAI database and performed a correlation analysis between ISYNA1 expression and infiltration levels of 24 immune cells.

### Correlation Between ISYNA1 Expression and Half Maximal Inhibitory Concentration (IC50) of Anticancer Drugs

We downloaded the RNA expression profiles of cancer cell lines and IC50 information of 192 anticancer drugs from the GDSC database (https://www.cancerrxgene.org/). Then, we calculated the association between ISYNA1 expression and IC50 of anticancer drugs.

## Results

### Expression of ISYNA1 in Pan-Cancer

**FIGURE 1:**
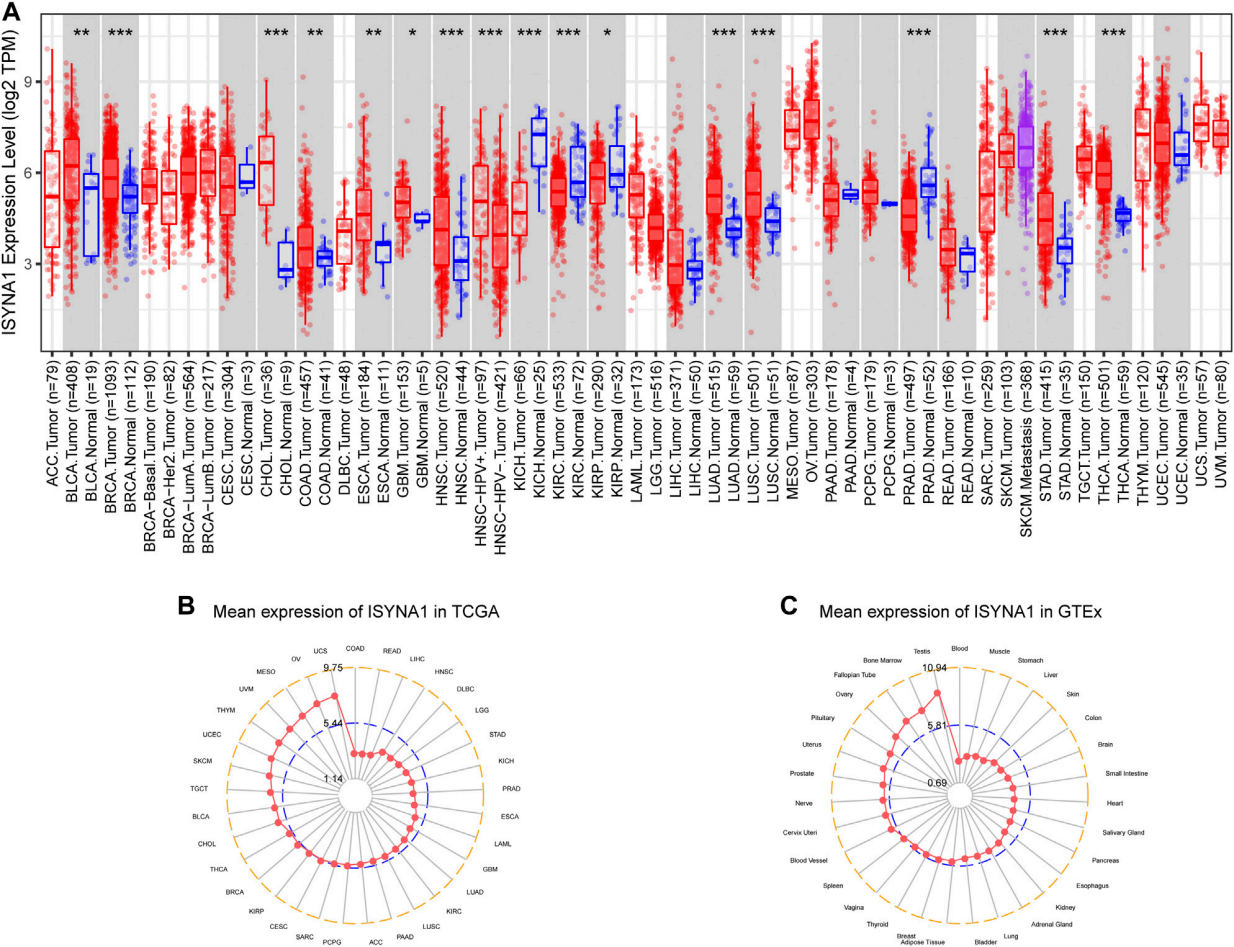
**(A)** Pan-cancer expression of ISYNA1. **(B)** ISYNA1 expression in tumor tissues from TCGA cohort. **(C)** ISYNA1 expression in normal tissues from GTEx cohort. *p < 0.05, **p < 0.01, ***p < 0.001, ****p < 0.0001.

First, we investigated ISYNA1 expression using the TIMER2 database. We found that ISYNA1 was highly expressed in 12 among 33 cancers types, including BLCA, BRCA, CHOL, COAD, ESCA, GBM, HNSC, LIHC, LUAD, LUSC, STAD, and THCA. In addition, ISYNA1 was lowly expressed in only four cancer types, such as KICH, KIRC, KIRP, and PRAD (Figure 1A). To evaluate the expression of ISYNA1 only in tumor tissues from TCGA cohort, we found that ISYNA1 expression was highest in tumor tissues of UCS and lowest in tumor tissues of COAD compared with other tumor tissues (Figure 1B). In normal tissues from GTEx database, we found that ISYNA1 expression was highest in testis tissue and lowest in blood (Figure 1C).

**FIGURE 2:**
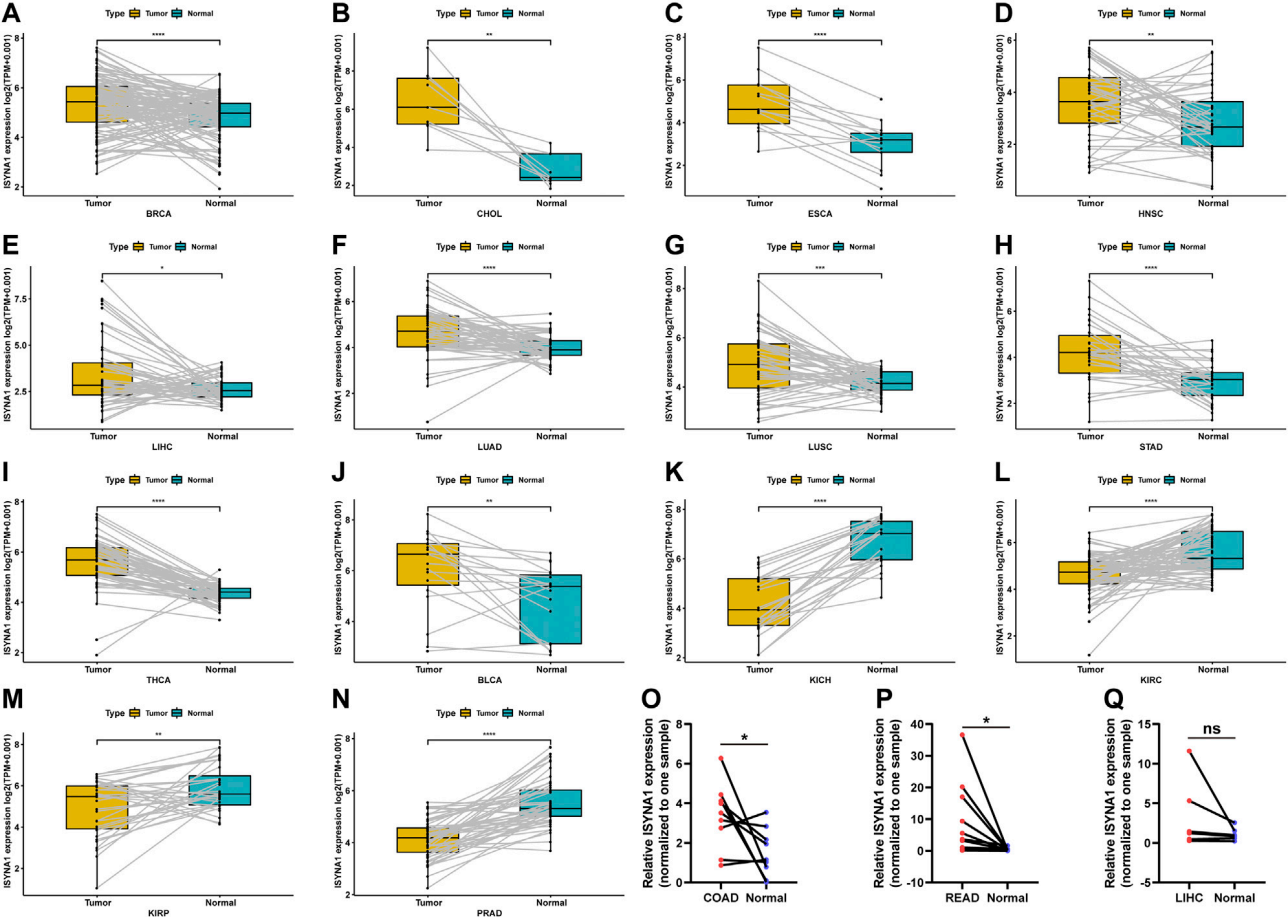
**(A–N)** ISYNA1 expression in paired tumor and adjacent normal tissues from TCGA in indicated tumor types. **(O–Q)** ISYNA1 expression in COAD, READ, and LIHC based on the qRT-PCR assay. *p < 0.05, **p < 0.01, ***p < 0.001, ****p < 0.0001.

Regarding the paired tumor and adjacent normal tissues in TCGA cohort, we observed that ISYNA1 was over-expressed in 10 cancers, such as BRCA, CHOL, ESCA, HNSC, LIHC, LUAD, LUSC, STAD, THCA, and BLCA (Figures 2A–J). In addition, ISYNA1 was lowly expressed in KICH, KIRC, KIRP, and PRAD (Figures 2K–N), which was consistent with the above results. In addition, we verified the expression of ISYNA1 by qRT-PCR in COAD, READ, and LIHC. Results revealed that ISYNA1 was highly expressed in COAD and READ (Figures 2O–Q).

**FIGURE 3:**
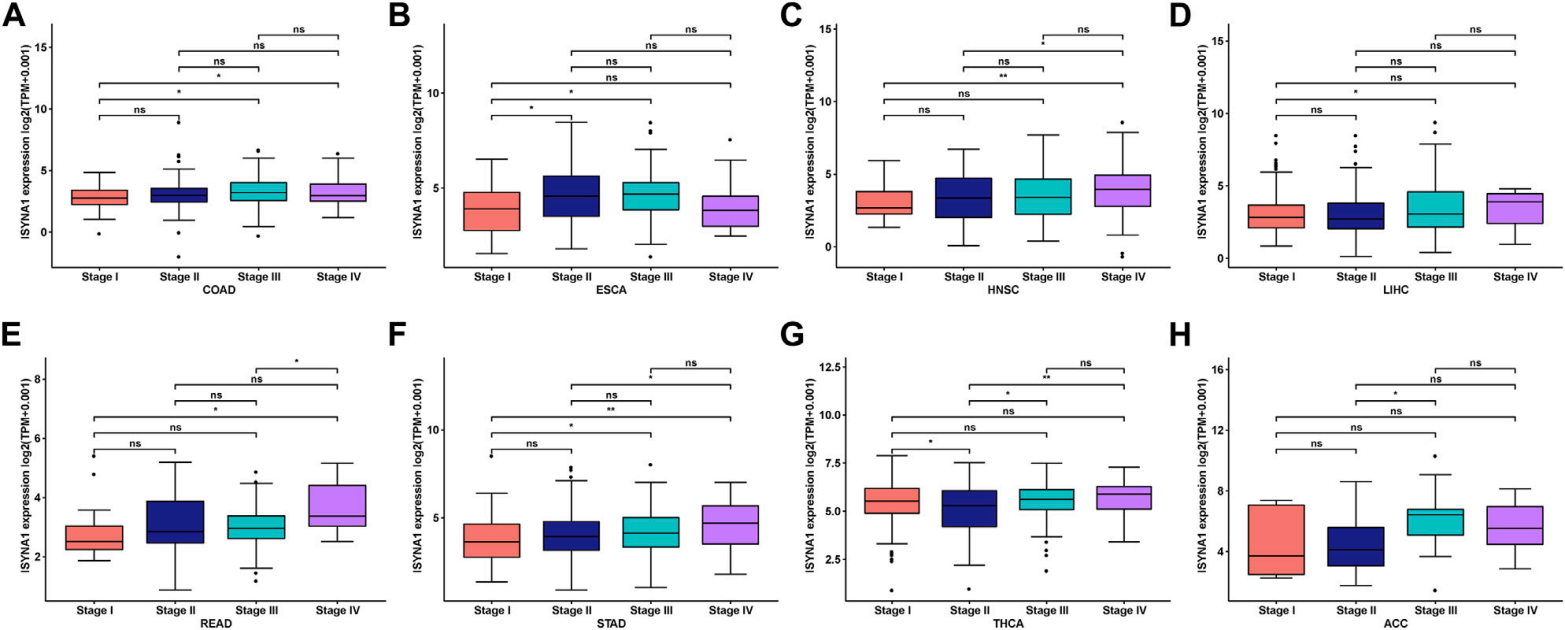
**(A–H)** ISYNA1 expression in various tumor stages in indicated tumor types. *p < 0.05, **p < 0.01, ***p < 0.001, ****p < 0.0001.

We further investigated ISYNA1 expression at different tumor stages. The results revealed that ISYNA1 expression was different in the various tumor stages in COAD, ESCA, HNSC, LIHC, READ, STAD, THCA, and ACC (Figures 3A–H).

### Gene Alteration of ISYNA1 in Pan-Cancer

**FIGURE 4:**
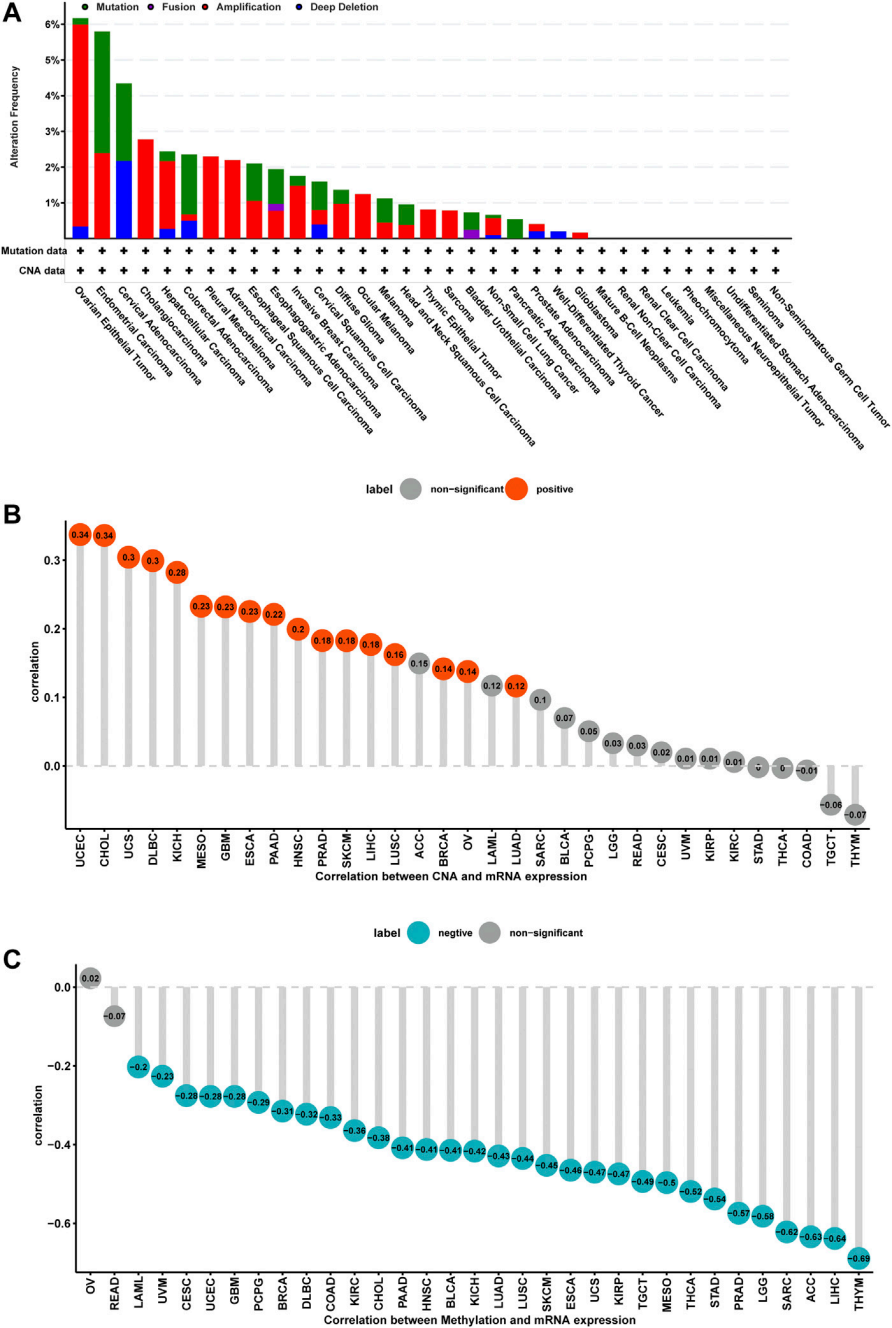
Gene alteration of ISYNA1. **(A)** The mutation and CNA status of ISYNA1 in TCGA pan-cancer. **(B)** The correlation between ISYNA1 expression and CNA. **(C)** The correlation between ISYNA1 expression and DNA methylation.

Mutation, DNA methylation, and CNA are main factors that influence gene expression. Thus, we assessed the mutations, DNA methylation, and CNA of ISYNA1. We revealed that the highest alteration frequency of ISYNA1 (>6%) was observed in OV patients, in which the “amplification” was the primary type (Figure 4A). For the correlation between ISYNA1 and CNA, we found that ISYNA1 expression was positively correlated with CNA in 17 among 33 cancers types (Figure 4B), indicating that the high CNA was one of the main causes of high ISYNA1 expression in pan-cancer, except in COAD. In addition, we also found that the promoter methylation level of ISYNA1 was negatively correlated with ISYNA1 expression in 31 among 33 cancers types (Figure 4C), including COAD (r = −0.33), indicating that the promoter methylation level of ISYNA1 mainly affects ISYNA1 mRNA expression in OCAD.

### Prognostic Value of ISYNA1

**FIGURE 5:**
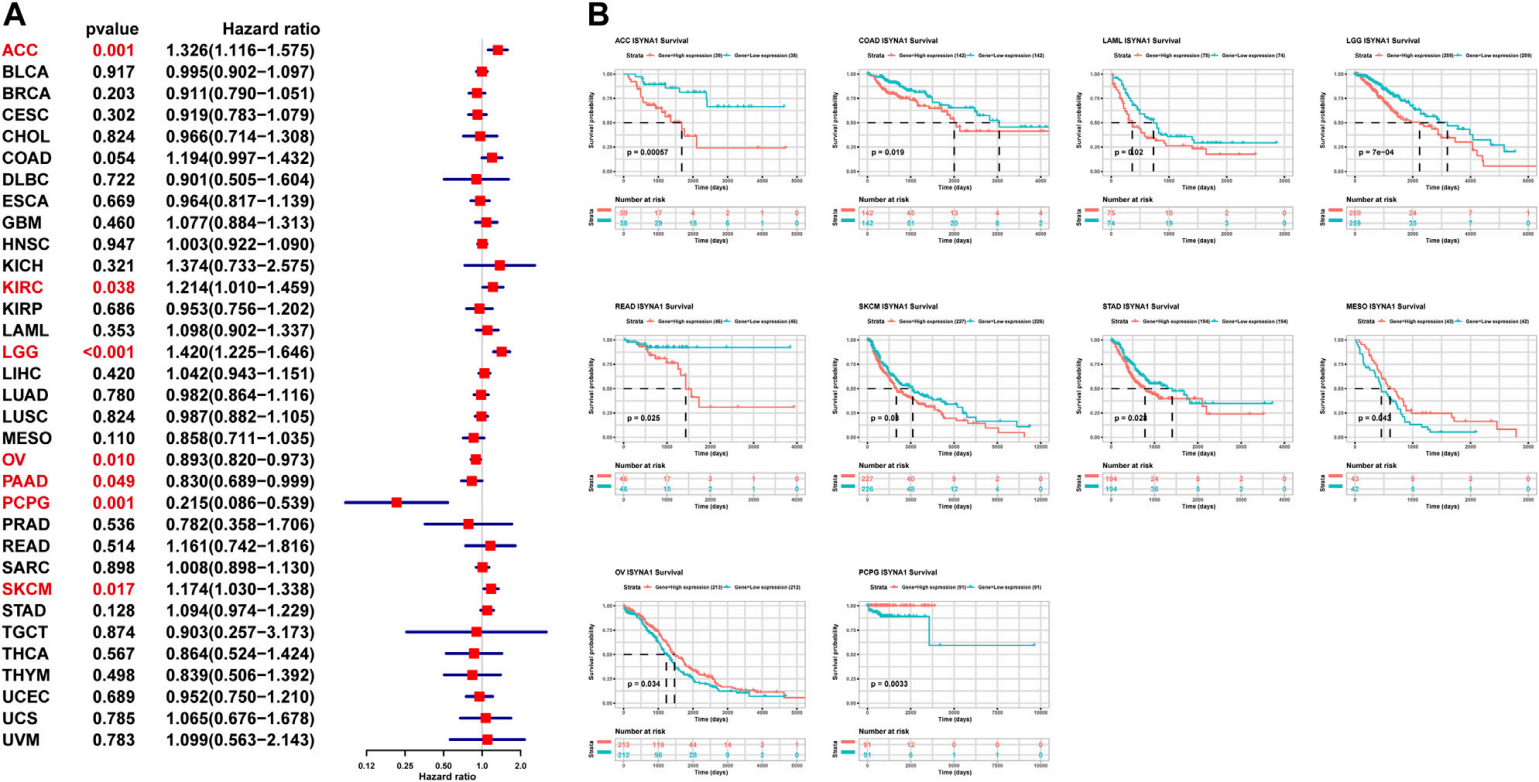
Prognostic value of ISYNA1. **(A)** The UniCox OS analysis of ISYNA1 in TCGA pan-cancer. Red represents significant results (*p* < 0.05). **(B)** Kaplan–Meier OS analysis of ISYNA1 in TCGA pan-cancer in indicated tumor types.

To assess the prognostic value of ISYNA1 in pan-cancer, the univariate Cox regression analysis (UniCox) and Kaplan–Meier survival analysis were conducted. Results of the UniCox indicated that ISYNA1 acts as a risk factor for overall survival (OS) of patients with ACC, KIRC, LGG, and SKCM, and acts as a protective factor for OS in patients with OV, PAAD, and PCPG (Figure 5A). The Kaplan–Meier OS analysis proved that the elevated expression of ISYNA1 predicted worse OS of patients with ACC, COAD, LAML, LGG, READ, SKCM, and STAD. In contrast, a high ISYNA1 expression predicted longer OS time in patients with MESO, OV, and PCPG (Figure 5B).

**FIGURE 6:**
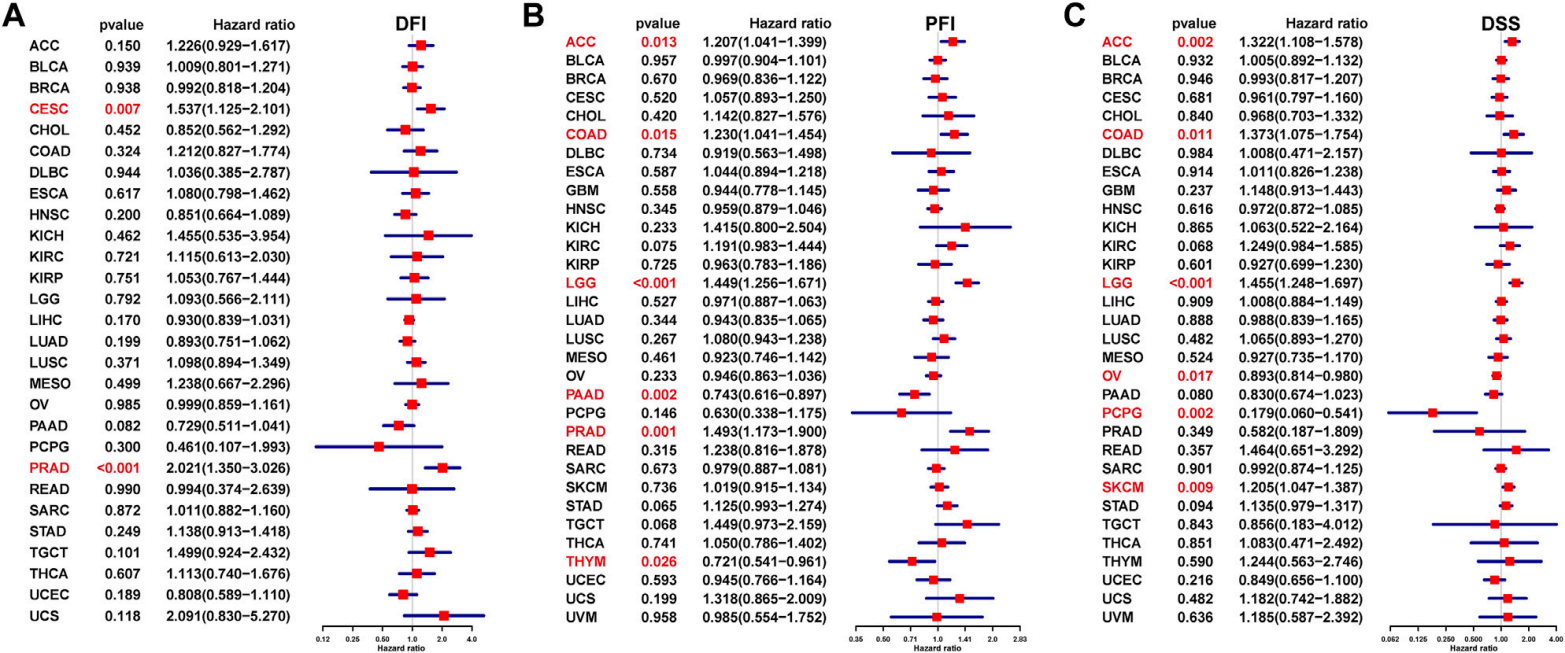
Prognostic value of ISYNA1 in predicting DFI, PFI, and DSS. **(A)** The UniCox results of DFI were shown using forest plots. **(B)** The UniCox results of PFI were shown using forest plots. **(C)** The UniCox results of DSS were shown using forest plots.

We also assessed the prognostic value of ISYNA1 in predicting disease-free interval (DFI), progression-free interval (PFI), and disease-specific survival (DSS) of tumor patients using univariate Cox regression analysis. For DFI, a high ISYNA1 expression predicted shorter DFI times in patients with CESC and PRAD (Figure 6A). For PFI, a high ISYNA1 expression predicted shorter PFI times in patients with ACC, COAD, LGG, and PRAD, and longer PFI time in patients with PAAD and THYM (Figure 6B). For DSS, a high ISYNA1 expression predicted a worse DSS status in patients with ACC, COAD, LGG, and SKCM, and a better DSS status in patients with OV and PCPG (Figure 6C).

### GSEA of ISYNA1

**FIGURE 7:**
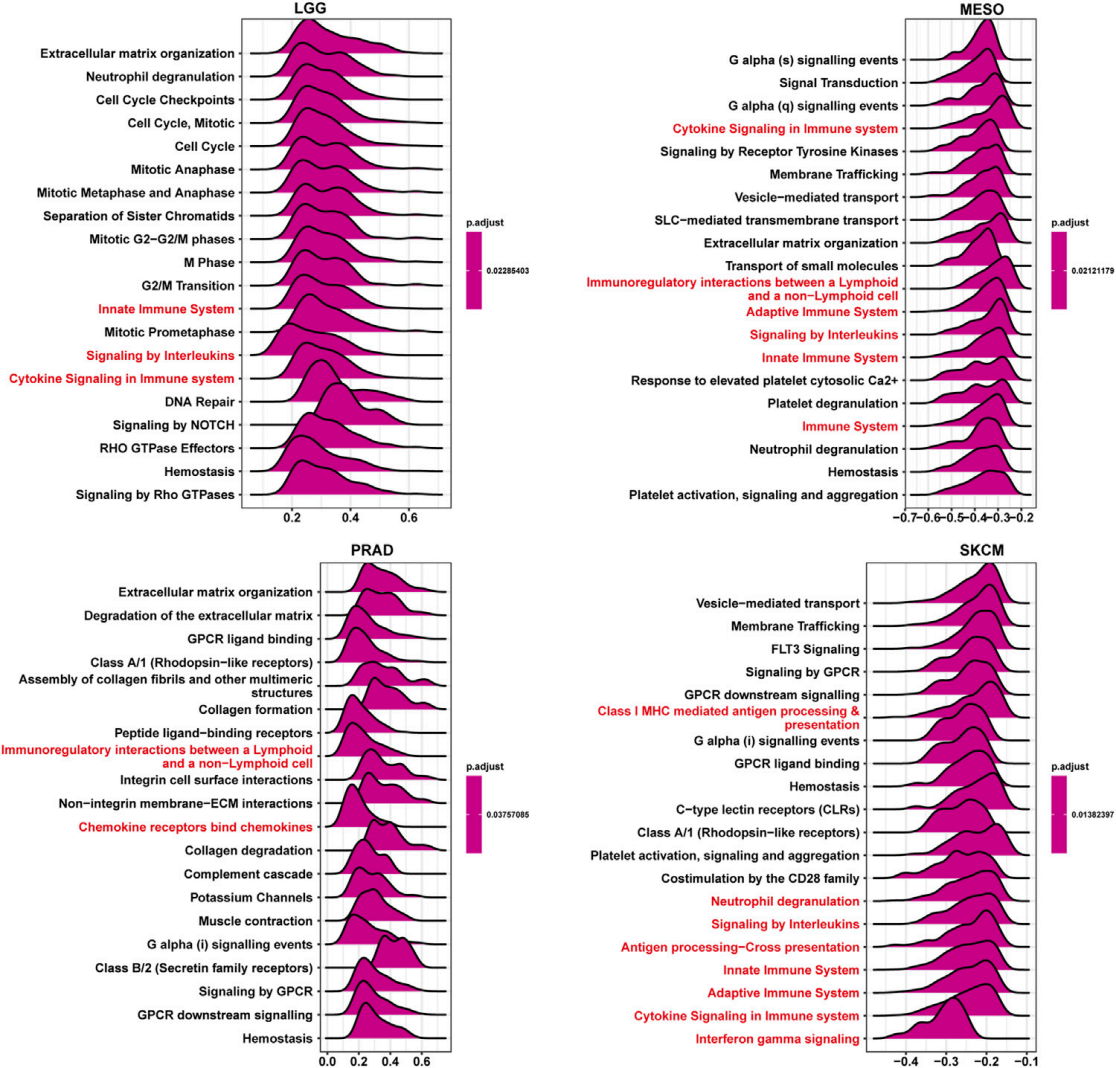
GSEA of ISYNA1. The top 20 GSEA results were shown in indicated tumor types. Red represents immune-related pathways.

To explore the potential pathways for the potential involvement of ISYNA1 in tumor progression, we further conducted the GSEA of ISYNA1 using TCGA pan-cancer data. Interestingly, we found common GSEA results for various tumor types. The immune regulation relevant pathways, such as “adaptive immune system,” “innate immune system,” “signaling by interleukins,” and “cytokine signaling in immune system,” were simultaneously enriched in LGG, MESO, PRAD, and SKCM (Figure 7).

### Relationship Between ISYNA1 Expression and Immune Cell Infiltration

**FIGURE 8:**
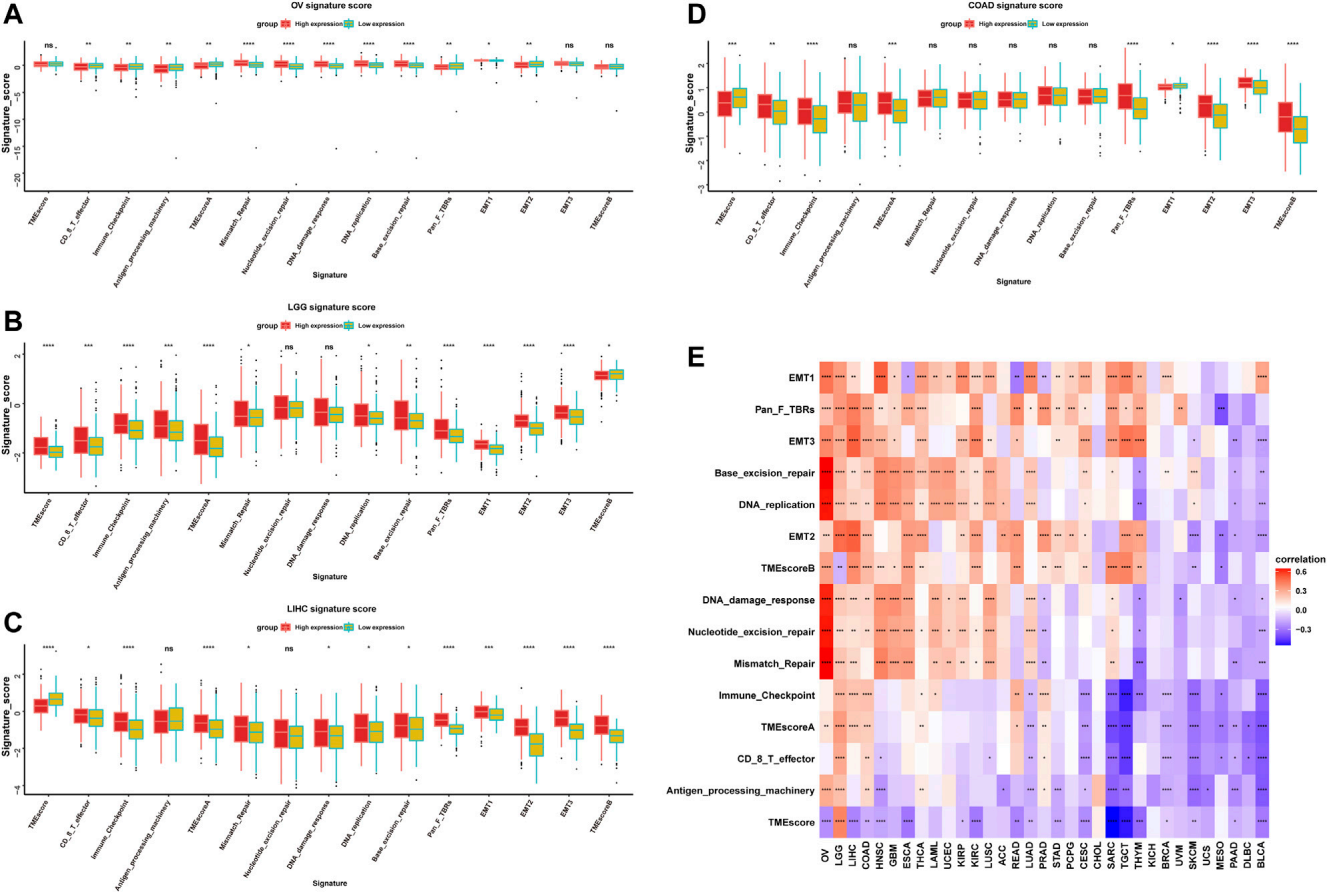
TME-related pathway analysis. **(A–D)** The scores of TME-related pathways in high- and low-ISYNA1 groups in indicated tumor types. **(E)** The correlation analysis between ISYNA1 expression and TME-related pathways in pan-cancer. Red represents positive correlation, and blue represents negative correlation. The darker the color, the stronger the correlation.

**FIGURE 9:**
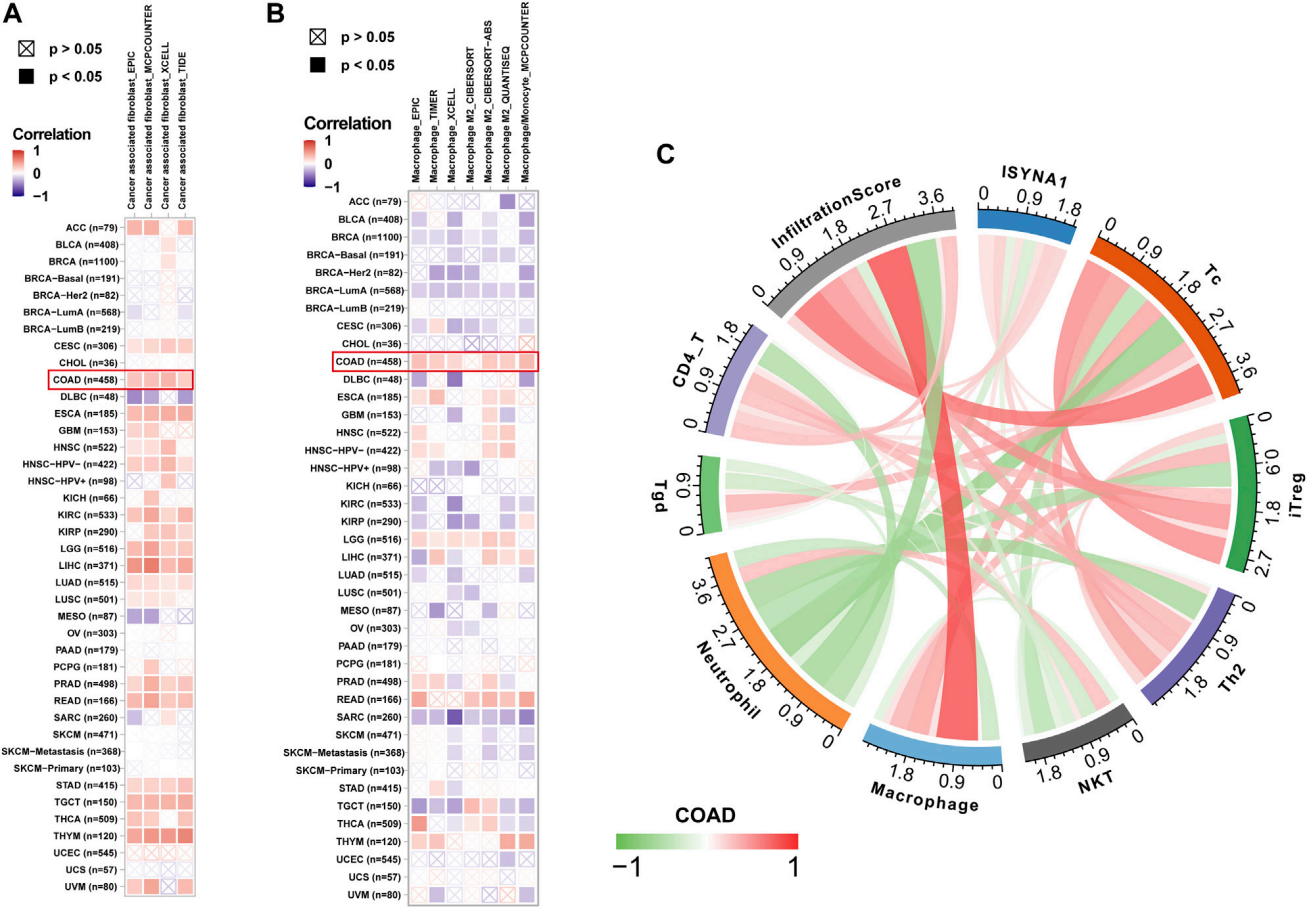
Immune infiltration analysis. **(A)** The relationship between ISYNA1 expression and infiltration levels of CAFs in pan-cancer using the TIMER2 database. **(B)** The relationship between ISYNA1 expression and infiltration levels of TAMs in pan-cancer using the TIMER2 database. **(C)** The correlation between ISYNA1 expression and infiltration levels of 22 immune cells using data from the ImmuCellAI database. Significant results were shown (correlation *p* < 0.05).

**FIGURE 10:**
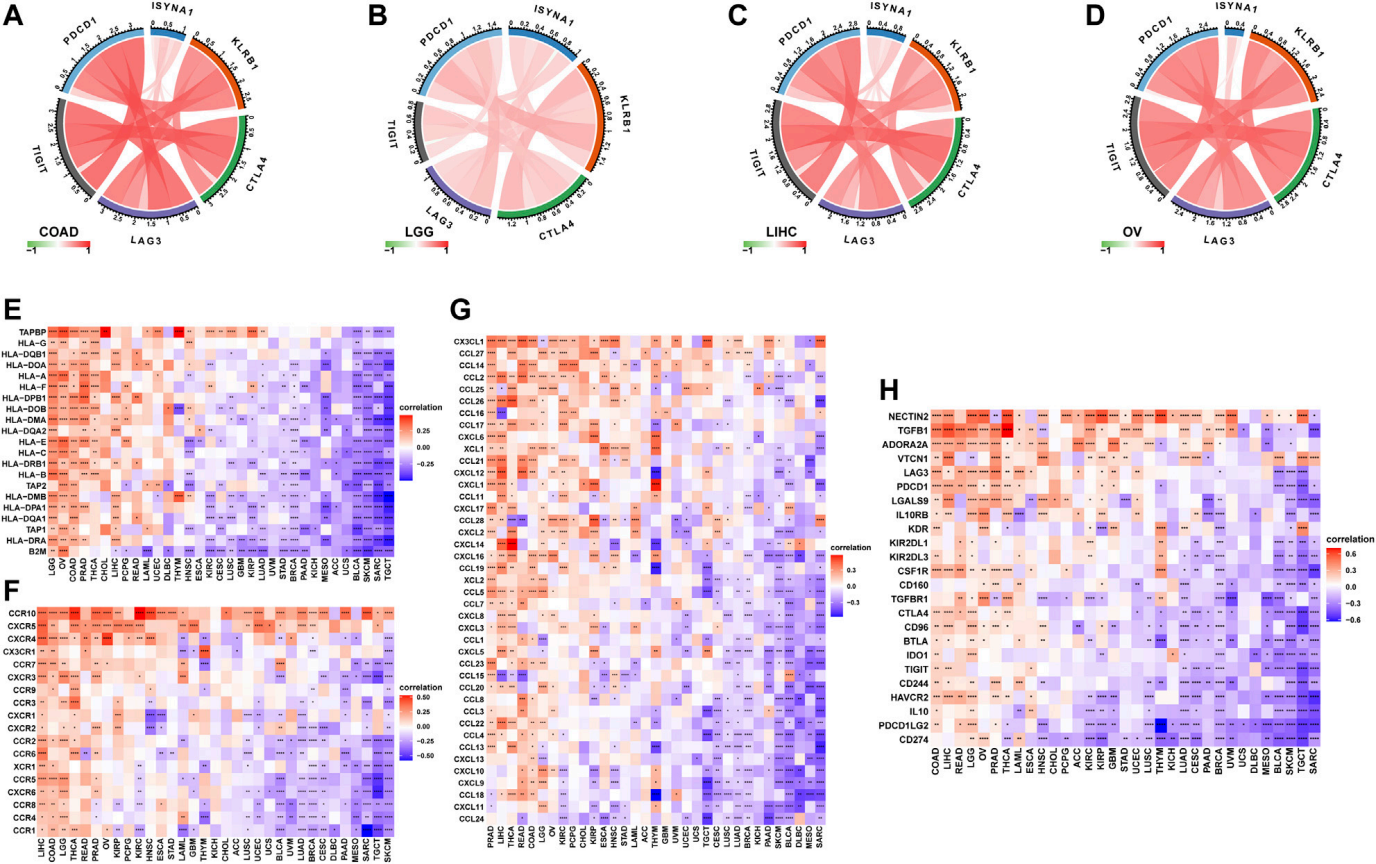
Correlation between ISYNA1 expression and immunosuppressive genes. **(A–D)** The correlation between ISYNA1 expression and immune checkpoints. Red lines represent positive correlation, and blue lines represent negative correlation. The darker the color, the stronger the correlation. **(E)** The correlation between ISYNA1 expression and MHC genes in pan-cancer. **(F)** The correlation between ISYNA1 expression and chemokine receptors in pan-cancer. **(G)** The correlation between ISYNA1 expression and chemokines in pan-cancer. **(H)** The correlation between ISYNA1 expression and immunosuppressive genes in pan-cancer. Red represents positive correlation, and blue represents negative correlation. The darker the color, the stronger the correlation. *p < 0.05, **p < 0.01, ***p < 0.001, ****p < 0.0001.

We further analyzed the role of ISYNA1 in TME. Through difference analysis and correlation analysis, we found that ISYNA1 was positively correlated with TME-related pathways mainly in OV, LGG, LIHC, and COAD (Figures 8A–E). For example, the scores of immune system-related pathways and tumor stroma-related pathways were higher in the high-ISYNA1 expression group in COAD (Figure 8E). By analyzing the association between ISYNA1 expression and immune cell infiltration using the TIMER2 database, we found that ISYNA1 expression was positively associated with the infiltration of CAFs and TAMs in COAD (Figures 9A,B). Using immune cell infiltration data from the ImmuCellAI database, we found that ISYNA1 expression was positively correlated with immunosuppressive cells, such as TAMs and iTreg (Figure 9C). These results indicated that high ISYNA1 expression may contribute to the tumor immunosuppressive microenvironment. To further explore the relationship between ISYNA1 and TIME, we conducted a correlation analysis among immunosuppressive genes, immune checkpoints, and ISYNA1. The results showed that ISYNA1 expression was positively correlated with immune checkpoints, such as PDCD1, TIGIT, LAG3, CTLA4, and KLRB1, in COAD, LGG, LIHC, and OV (Figures 10A–D). We also observed that ISYNA1 was positively correlated with MHC genes (Figure 10E), chemokines and chemokine receptors (Figures 10F,G), and immunosuppressive genes (Figure 10H) in COAD. Interestingly, there was no correlation between ISYNA1 and CD274 (PD-L1) expression, which was validated by qRT-PCR in COAD, READ, and LIHC (Supplementary Figures S1A–C). These results revealed that ISYNA1 expression predicted the immunosuppressive microenvironment.

### Correlation Analysis of ISYNA1 Expression and Drug Response

**FIGURE 11:**
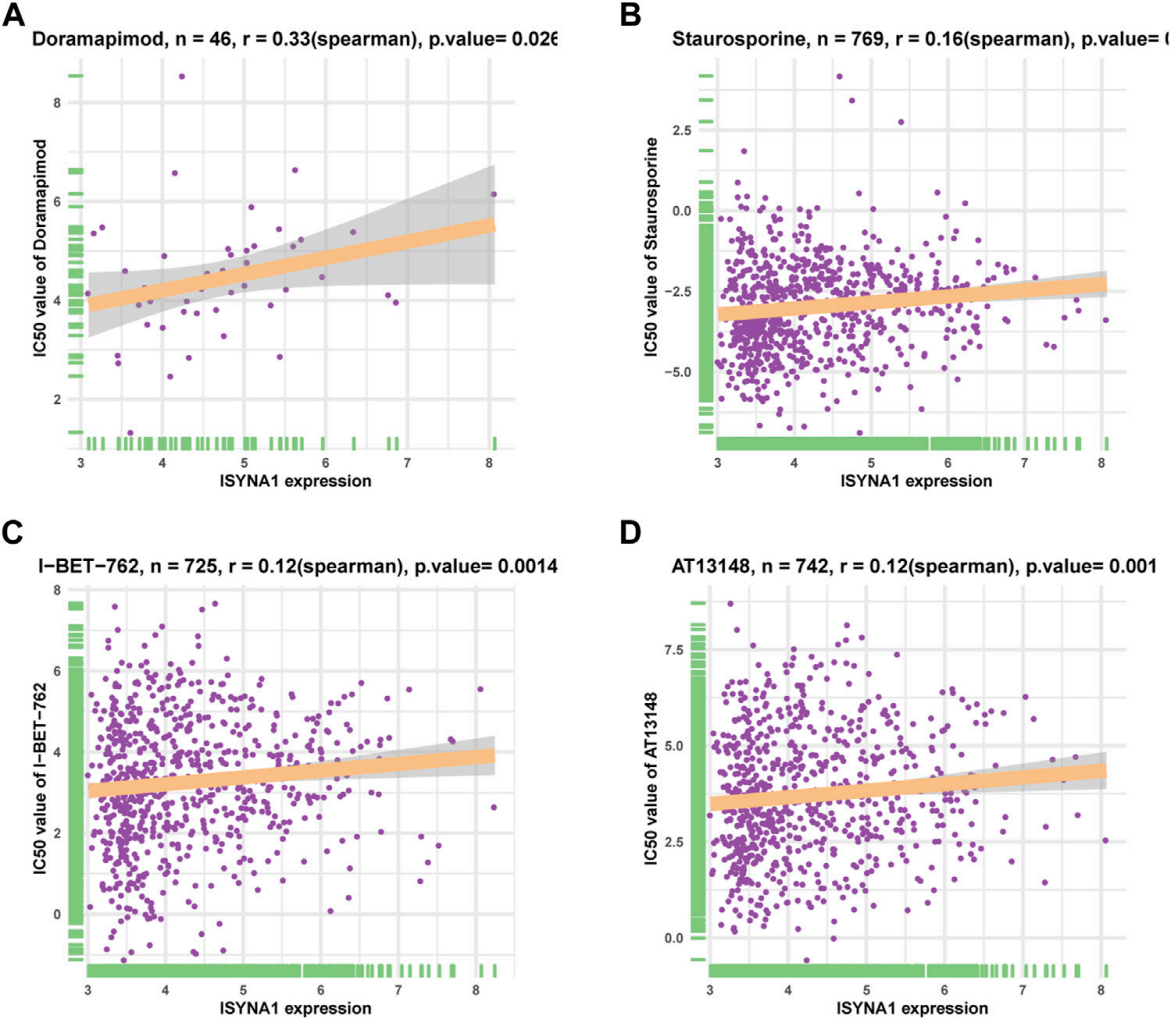
Correlation analysis of ISYNA1 expression and drug response. **(A–D)** The correlation between ISYNA1 expression and IC50 of indicated anticancer drugs.

We downloaded half-inhibitory concentration (IC50) values of 192 drugs and gene expression profiles of 809 cell lines from the Genomics of Drug Sensitivity in Cancer database (GDSC: https://www.cancerrxgene.org/) and analyzed Spearman’s correlation between ISYNA1 expression and IC50 values. Among 192 anticancer drugs, the IC50 values of 26 drugs were positively correlated with ISYNA1 expression, while only the IC50 values of three drugs were negatively correlated with ISYNA1 expression (Supplementary Table S1, Figures 11A–D). These results revealed that patients with high ISYNA1 expression were predicted to be resistant to most anticancer drugs.

## Discussion

COAD is one of the most common cancers and is a fatal factor in the health of humans globally (Simon, 2016; Thanikachalam and Khan, 2019). A body of studies have shown that the reconstruction of TIME by colorectal cancer cells plays a key role in the progression of COAD, weakening the response of patients with COAD to the treatment and leading to worse survival status (Becht et al., 2016; Franke et al., 2019; Ganesh et al., 2019; Lichtenstern et al., 2020). Thus, the identification of essential genes that could affect TIME in COAD is urgently needed.

ISYNA1 encodes the rate-limiting enzyme inositol-3-phosphate synthase. Until now, there are few studies on ISYNA1 in tumor research. It was reported that ISYNA1 functions as an oncogene in several tumors (Azimi et al., 2014). For example, ISYNA1 was observed as highly expressed in BLCA, regulating the proliferation and apoptosis of BLCA cells (Guo et al., 2019). In pancreatic cancer, the ISYNA1-p21/ZEB-1 pathway could contribute to tumor progression (Zhou L et al., 2020).

In our study, we first assessed the expression of ISYNA1 and found the elevated ISYNA1 expression in 12 among 33 cancers types, including BLCA, BRCA, CHOL, COAD, ESCA, GBM, HNSC, LIHC, LUAD, LUSC, STAD, and THCA. In addition, decreased ISYNA1 expression was observed in four cancer types, such as KICH, KIRC, KIRP, and PRAD. To determine the prognostic value of ISYNA1, we conducted the UniCox and Kaplan–Meier survival analysis in TCGA cohort. Results of the UniCox indicated that ISYNA1 acts as a risk factor for OS in patients with ACC, KIRC, LGG, and SKCM and acts as a protective factor for OS in patients with OV, PAAD, and PCPG. The Kaplan–Meier OS analysis revealed that an elevated ISYNA1 expression indicated worse survival of patients with ACC, COAD, LAML, LGG, READ, SKCM, and STAD. In contrast, a high ISYNA1 expression predicted longer OS time in patients with MESO, OV, and PCPG.

GSEA results predicted the immune regulatory function of ISYNA1 in COAD. Through the analysis of the correlation between ISYNA1 expression and TME, we found that the scores of immune system-related pathways and tumor stroma-related pathways were higher in the high-ISYNA1 expression group in COAD. Furthermore, the association among immune cell infiltration, stroma cell infiltration, and ISYNA1 expression was analyzed. Results revealed that the infiltration levels of TAMs and CAFs were positively correlated with ISYNA1 expression in COAD. We further validated the results using immune cell infiltration data from the ImmuCellAI database. The results also revealed that ISYNA1 expression was positively associated with the infiltration of TAMs in COAD. Moreover, we found that ISYNA1 expression was positively correlated with iTreg cells in COAD. Through the correlation analysis between ISYNA1 expression and immunosuppressive genes, we found that ISYNA1 expression was positively correlated with most immunosuppressive genes and immune checkpoints in COAD, such as TGFBR1, IL10, IL10RB, CTLA4, CD274, PDCD1, TIGIT, and LAG3.

In conclusion, our findings revealed that ISYNA1 is an oncogene and a prognostic marker in COAD. High ISYNA1 expression may contribute to the immunosuppressive microenvironment in COAD. Targeting ISYNA1 may be a potential method for COAD therapy.

## Data Availability

The datasets presented in this study can be found in online repositories. The names of the repository/repositories and accession number(s) can be found in the article/Supplementary Material.

## Glossary

**ACC**: adrenocortical carcinoma
**BLCA**: bladder urothelial carcinoma
BRCA: breast invasive carcinoma
**CESC**: cervical squamous cell carcinoma and endocervical adenocarcinoma
**CHOL**: cholangiocarcinoma
**COAD**: colon adenocarcinoma
**DLBC**: lymphoid neoplasm diffuse large b-cell lymphoma
**ESCA**: esophageal carcinoma
**GBM**: glioblastoma multiforme
**HNSC**: head and neck squamous cell carcinoma
**KICH**: kidney chromophobe
**KIRC**: kidney renal clear cell carcinoma
**KIRP**: kidney renal papillary cell carcinoma
**LAML**: acute myeloid leukemia
**LGG**: lower-grade glioma
**LIHC**: liver hepatocellular carcinoma
**LUAD**: lung adenocarcinoma
**LUSC**: lung squamous cell carcinoma
**MESO**: mesothelioma
**OV**: ovarian serous cystadenocarcinoma
**PAAD**: pancreatic adenocarcinoma
**PCPG**: pheochromocytoma and paraganglioma
**PRAD**: prostate adenocarcinoma
**READ**: rectum adenocarcinoma
**SARC**: sarcoma
**SKCM**: skin cutaneous melanoma
**STAD**: stomach adenocarcinoma
**TGCT**: testicular germ cell tumor
**THCA**: thyroid carcinoma
**THYM**: thymoma
**UCEC**: uterine corpus endometrial carcinoma
**UCS**: uterine carcinosarcoma
**UVM**: uveal melanoma
