# Supplementary material for: ISYNA1: An Immunomodulatory-Related Prognostic Biomarker in Colon Adenocarcinoma and Pan-Cancer
Source: Front Cell Dev Biol. 2022 Feb 14;10:792564. doi: 10.3389/fcell.2022.792564 (PMC8883116; doi:10.3389/fcell.2022.792564)
Supplement: Supplementary file 1 [file DataSheet2.PDF]

Supplementary Figure and Figure legends

Supplementary Figure 1

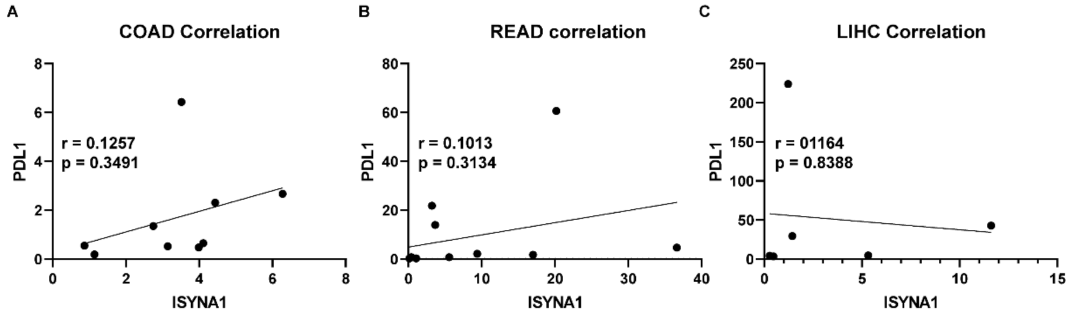

Supplementary Figure 1. The correlation between ISYNA1 and PDL1 (A-C). The correlation between ISYNA1 and PDL1 based on qRT-RCP results.
